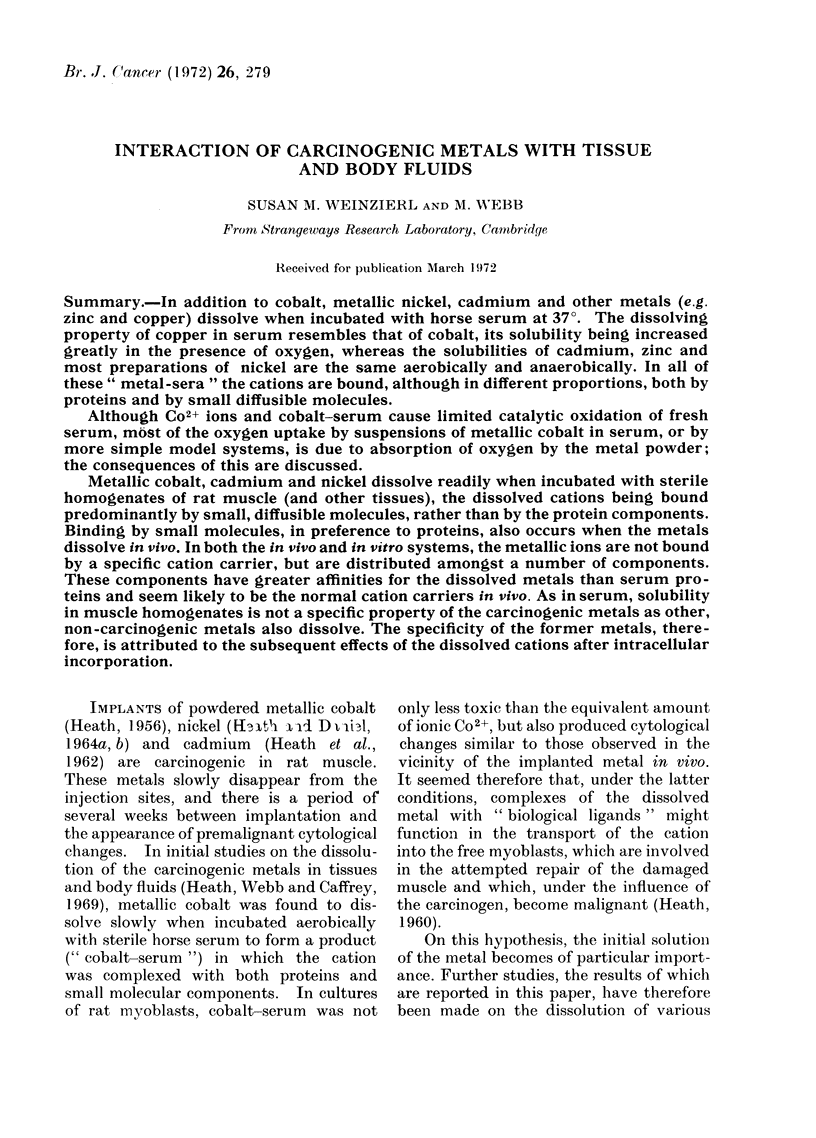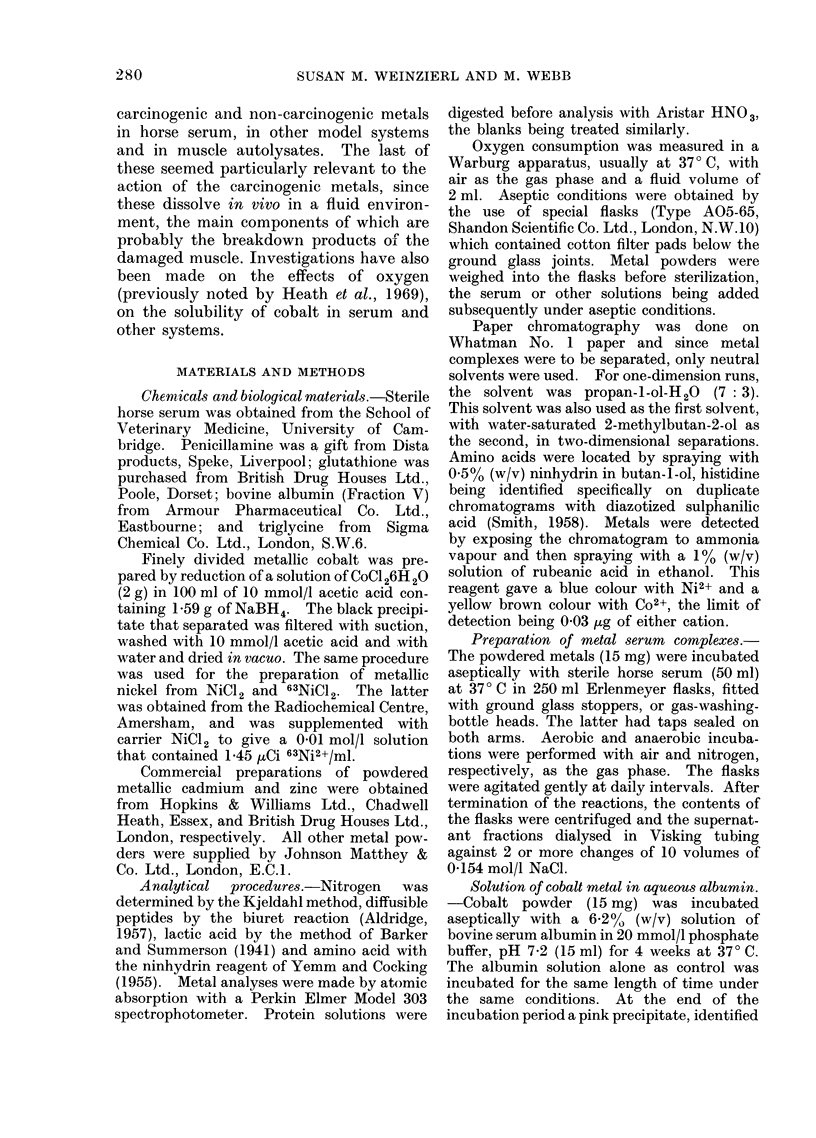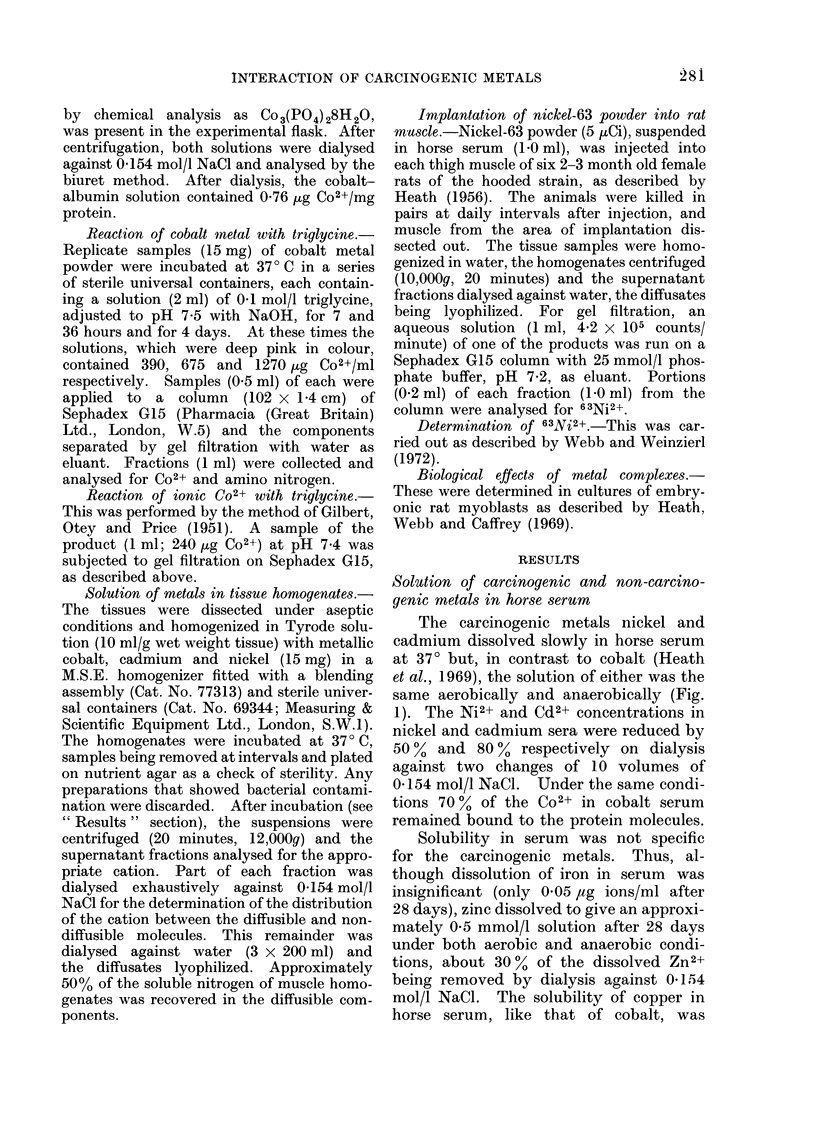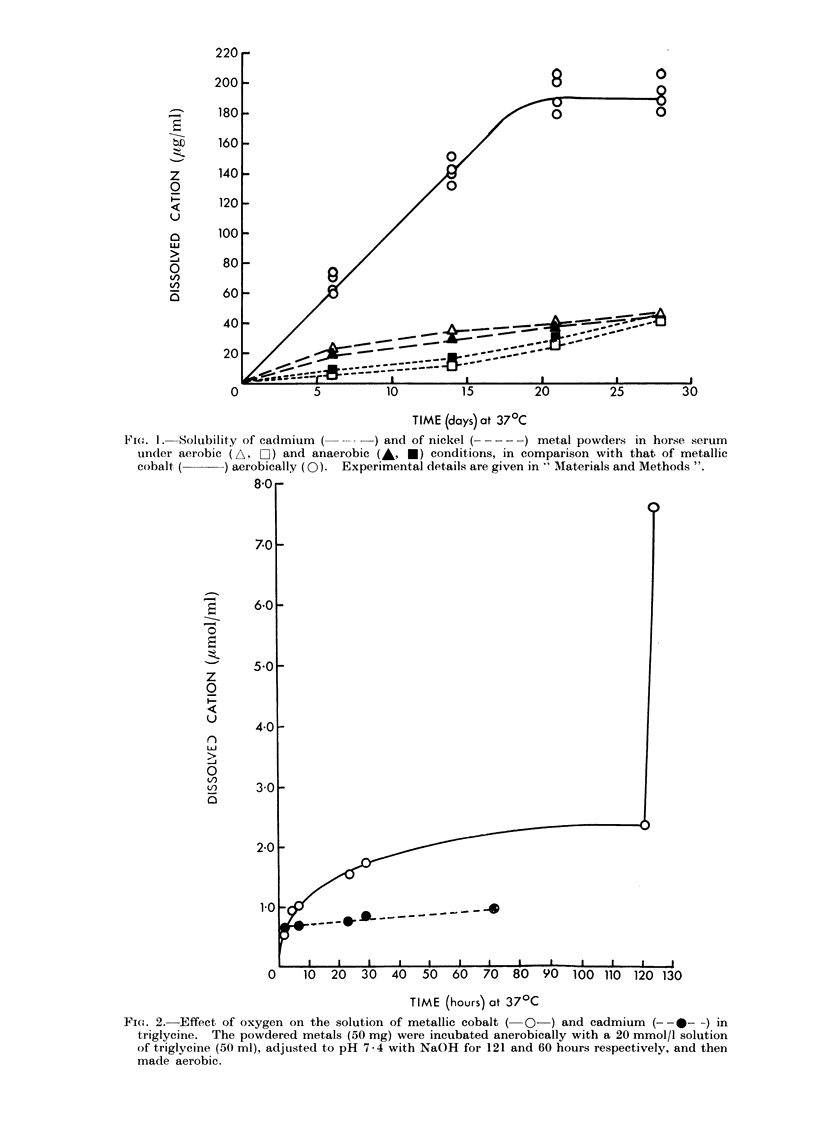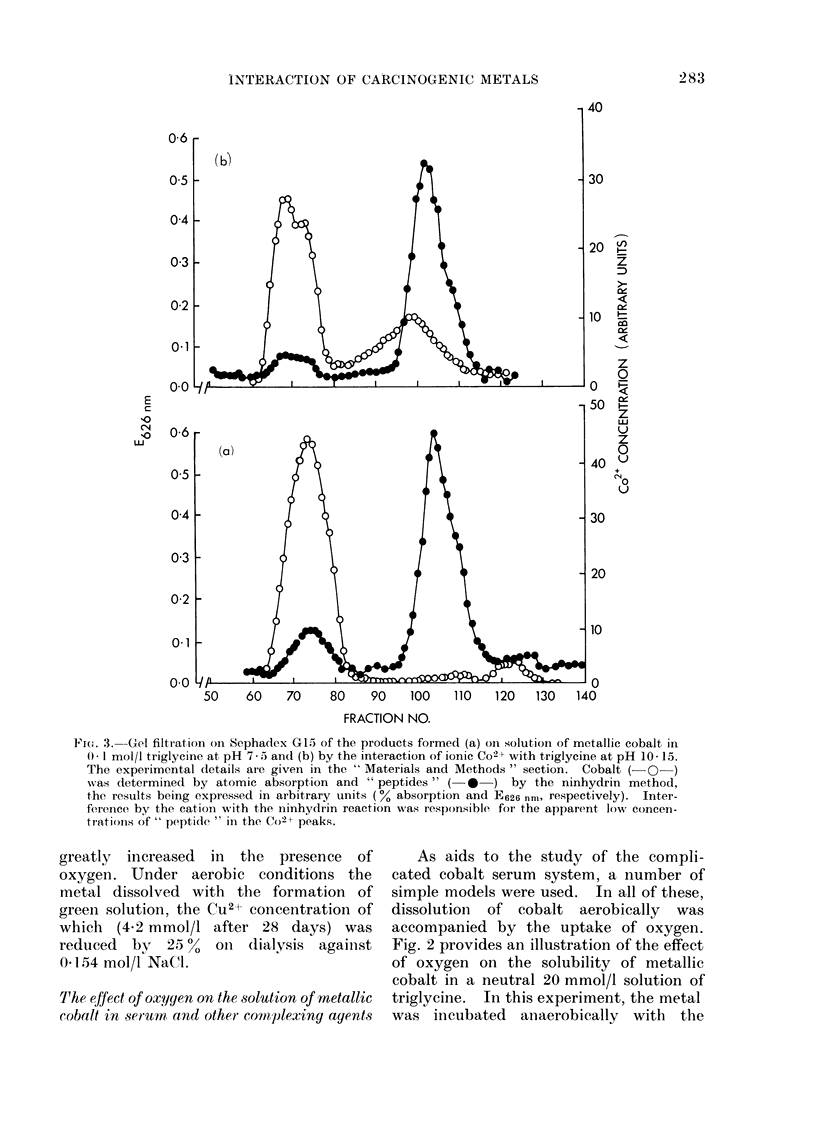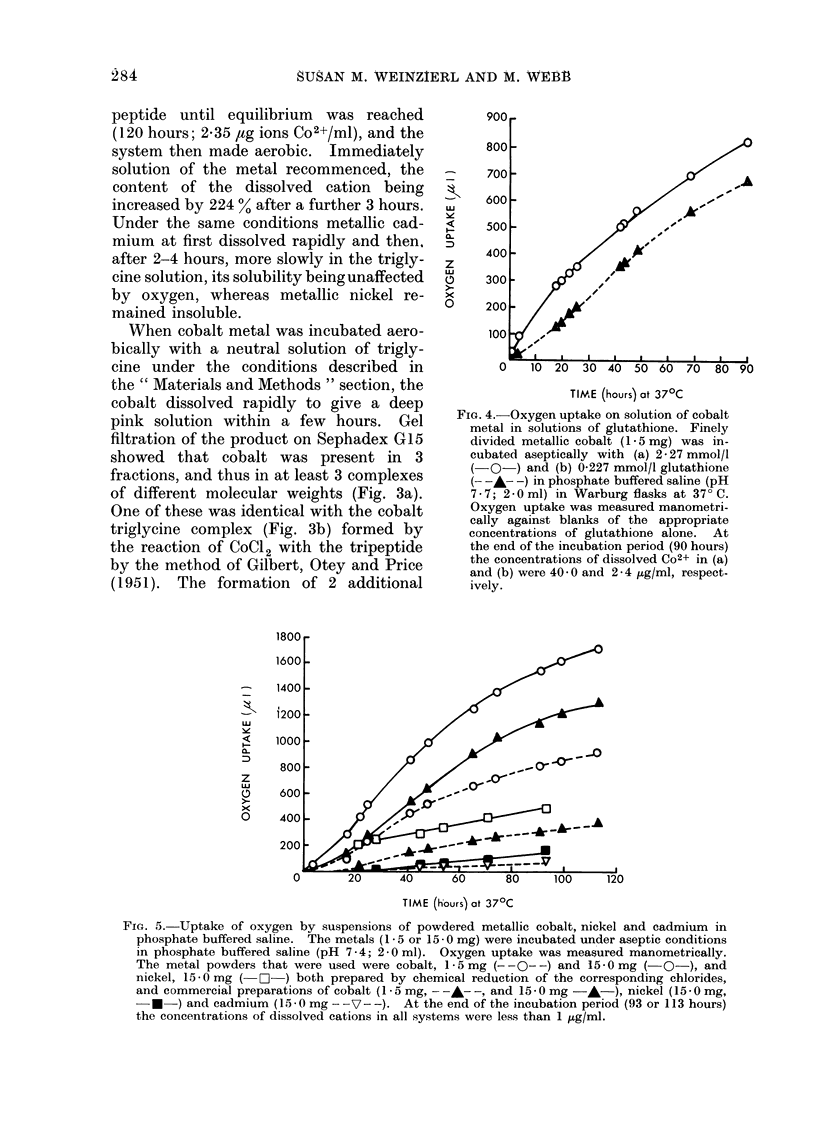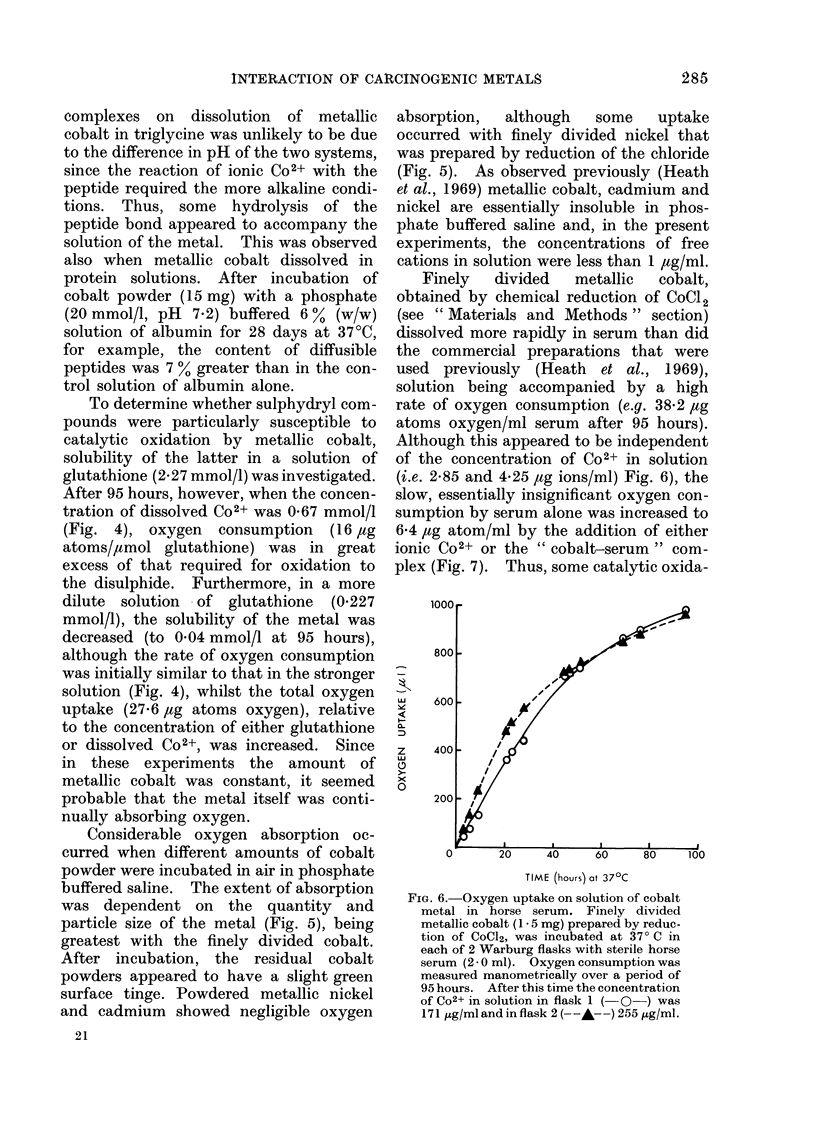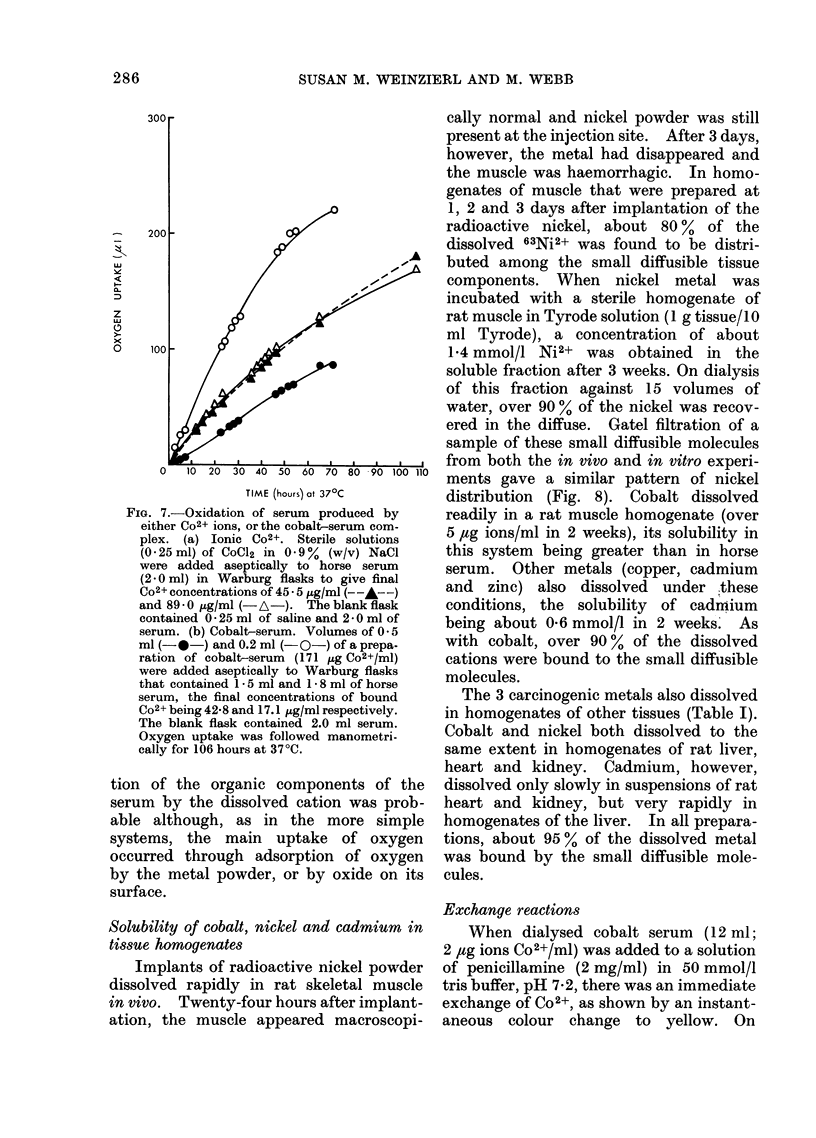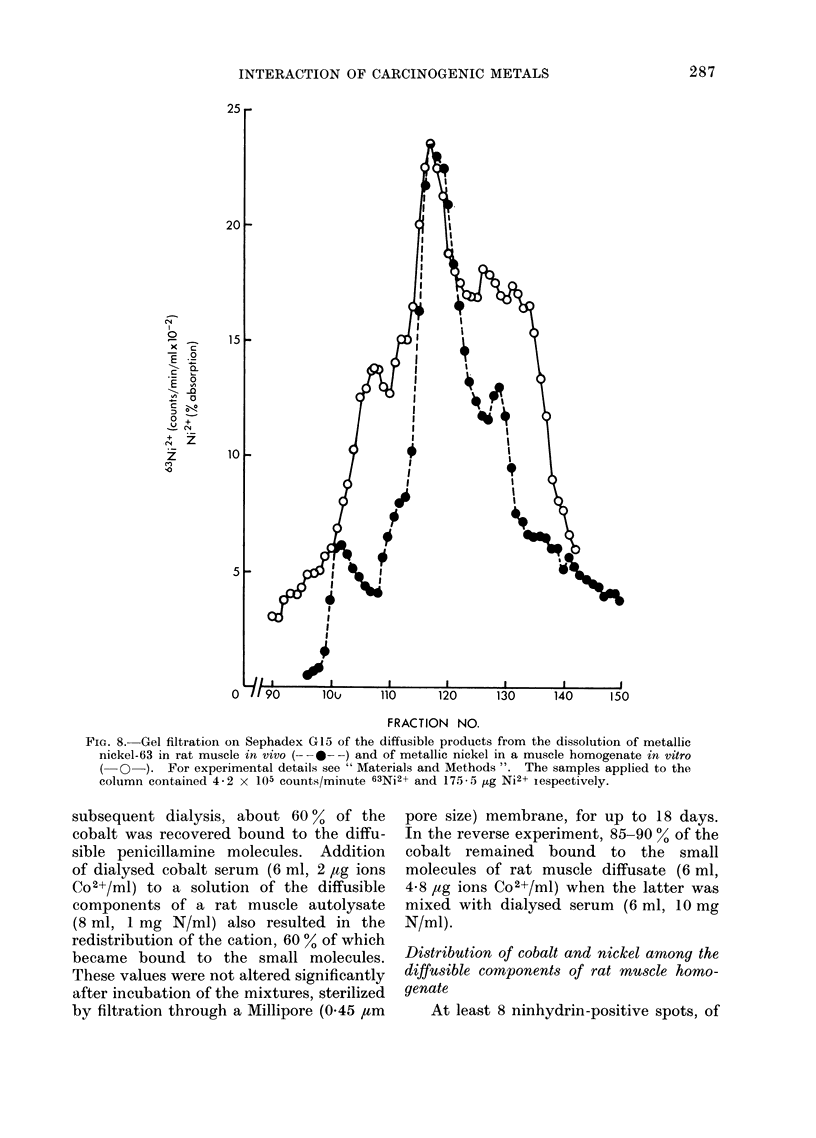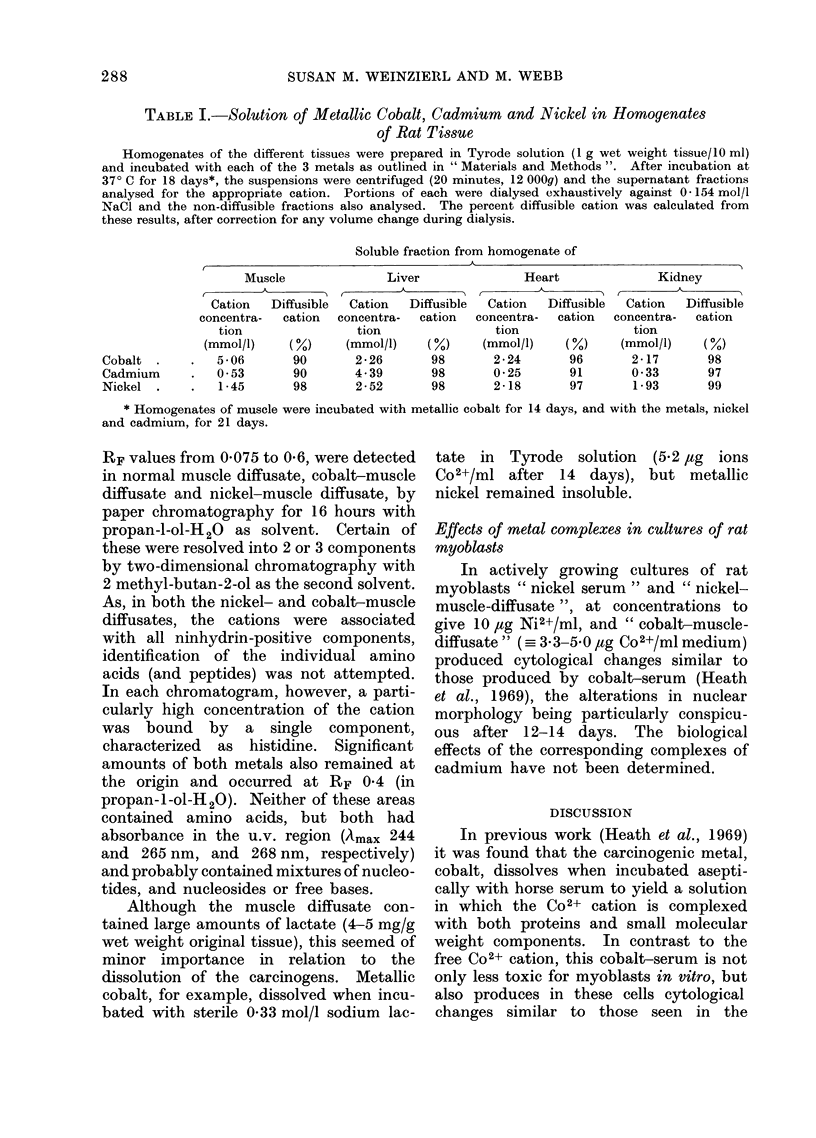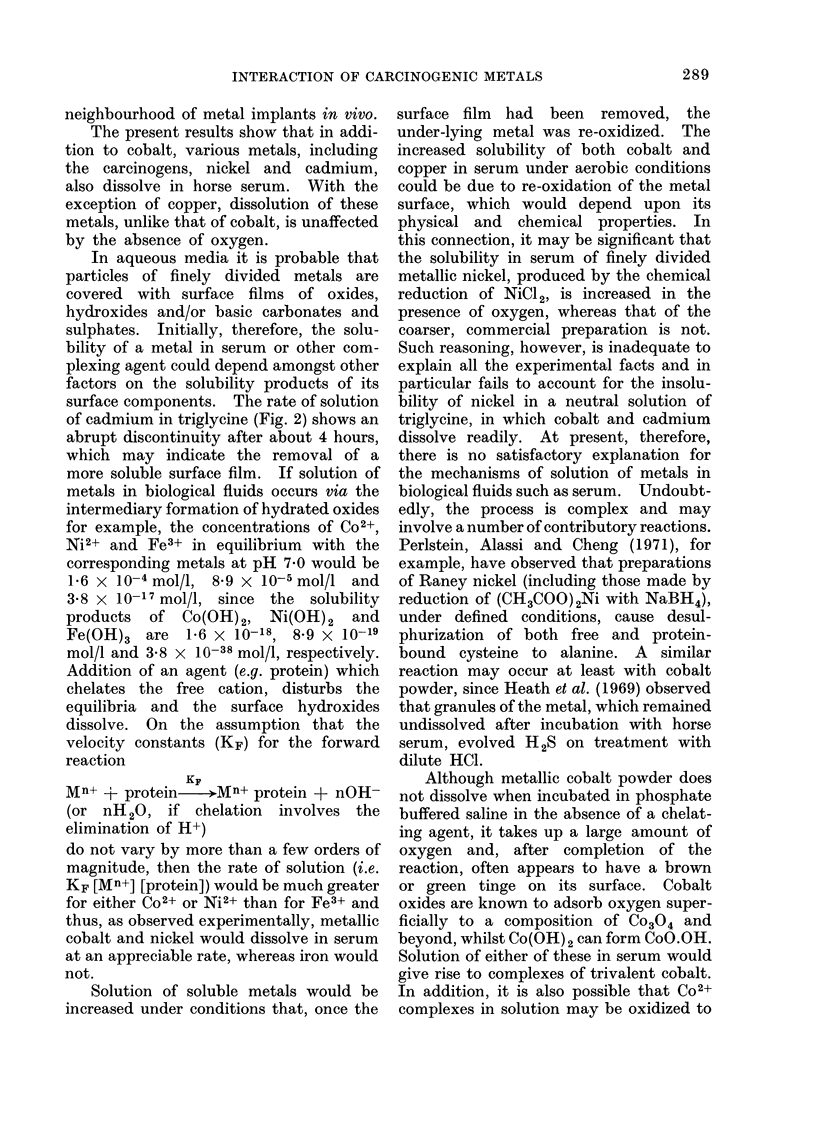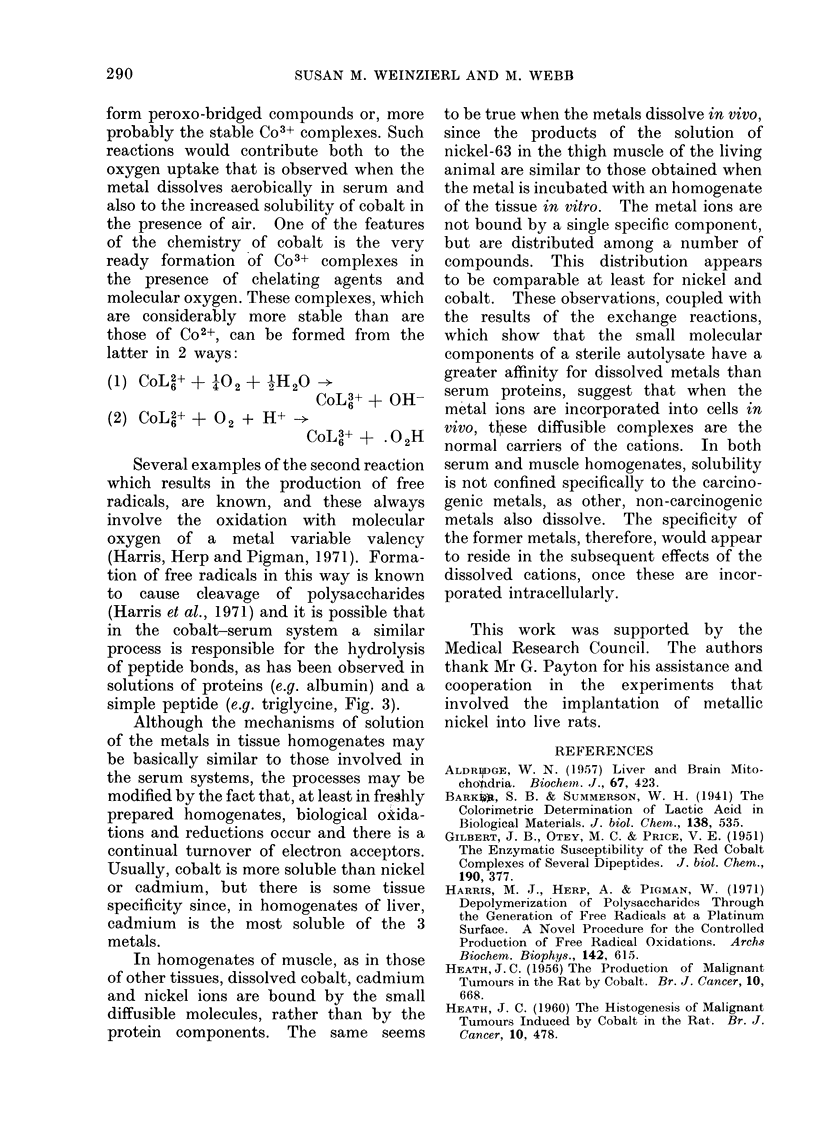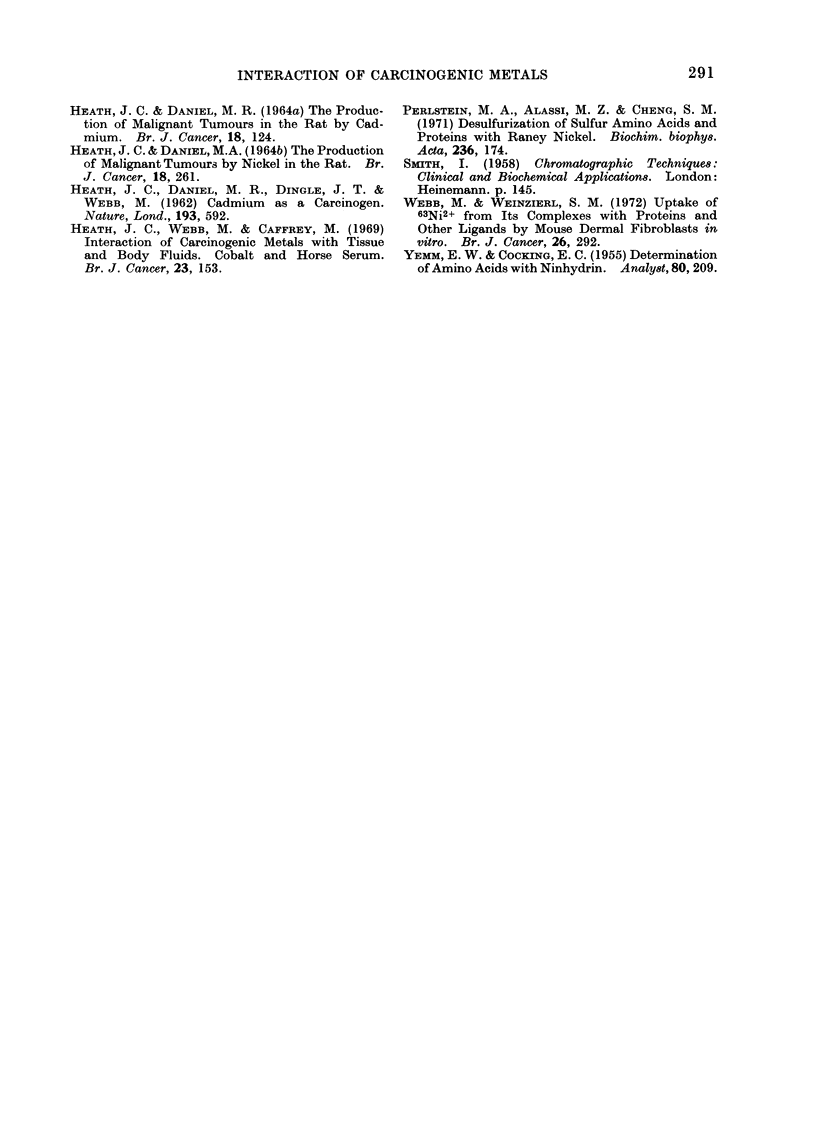# Interaction of Carcinogenic Metals with Tissue and Body Fluids

**DOI:** 10.1038/bjc.1972.38

**Published:** 1972-08

**Authors:** Susan M. Weinzierl, M. Webb

## Abstract

In addition to cobalt, metallic nickel, cadmium and other metals (*e.g.* zinc and copper) dissolve when incubated with horse serum at 37°. The dissolving property of copper in serum resembles that of cobalt, its solubility being increased greatly in the presence of oxygen, whereas the solubilities of cadmium, zinc and most preparations of nickel are the same aerobically and anaerobically. In all of these “metal-sera” the cations are bound, although in different proportions, both by proteins and by small diffusible molecules.

Although Co^2+^ ions and cobalt-serum cause limited catalytic oxidation of fresh serum, most of the oxygen uptake by suspensions of metallic cobalt in serum, or by more simple model systems, is due to absorption of oxygen by the metal powder; the consequences of this are discussed.

Metallic cobalt, cadmium and nickel dissolve readily when incubated with sterile homogenates of rat muscle (and other tissues), the dissolved cations being bound predominantly by small, diffusible molecules, rather than by the protein components. Binding by small molecules, in preference to proteins, also occurs when the metals dissolve *in vivo.* In both the *in vivo* and *in vitro* systems, the metallic ions are not bound by a specific cation carrier, but are distributed amongst a number of components. These components have greater affinities for the dissolved metals than serum proteins and seem likely to be the normal cation carriers *in vivo.* As in serum, solubility in muscle homogenates is not a specific property of the carcinogenic metals as other, non-carcinogenic metals also dissolve. The specificity of the former metals, therefore, is attributed to the subsequent effects of the dissolved cations after intracellular incorporation.


Br. J. Cancer (1972) 26, 279

### INTERACTION OF CARCINOGENIC METALS WITH TISSUE AND BODY FLUIDS

SUSAN M. WEINZIERL AND M. WEBB

*From Strangeways Research Laboratory, Cambridge*

Received for publication March 1972

**Summary.—In addition to cobalt, metallic nickel, cadmium and other metals (*e.g.* zinc and copper) dissolve when incubated with horse serum at 37°. The dissolving property of copper in serum resembles that of cobalt, its solubility being increased greatly in the presence of oxygen, whereas the solubilities of cadmium, zinc and most preparations of nickel are the same aerobically and anaerobically. In all of these " metal-sera " the cations are bound, although in different proportions, both by proteins and by small diffusible molecules.**

**Although $Co^{2+}$ ions and cobalt–serum cause limited catalytic oxidation of fresh serum, most of the oxygen uptake by suspensions of metallic cobalt in serum, or by more simple model systems, is due to absorption of oxygen by the metal powder; the consequences of this are discussed.**

**Metallic cobalt, cadmium and nickel dissolve readily when incubated with sterile homogenates of rat muscle (and other tissues), the dissolved cations being bound predominantly by small, diffusible molecules, rather than by the protein components. Binding by small molecules, in preference to proteins, also occurs when the metals dissolve *in vivo*. In both the *in vivo* and *in vitro* systems, the metallic ions are not bound by a specific cation carrier, but are distributed amongst a number of components. These components have greater affinities for the dissolved metals than serum proteins and seem likely to be the normal cation carriers *in vivo*. As in serum, solubility in muscle homogenates is not a specific property of the carcinogenic metals as other, non-carcinogenic metals also dissolve. The specificity of the former metals, therefore, is attributed to the subsequent effects of the dissolved cations after intracellular incorporation.**

IMPLANTS of powdered metallic cobalt (Heath, 1956), nickel (Heath and Daniel, 1964*a*, *b*) and cadmium (Heath *et al.*, 1962) are carcinogenic in rat muscle. These metals slowly disappear from the injection sites, and there is a period of several weeks between implantation and the appearance of premalignant cytological changes. In initial studies on the dissolution of the carcinogenic metals in tissues and body fluids (Heath, Webb and Caffrey, 1969), metallic cobalt was found to dissolve slowly when incubated aerobically with sterile horse serum to form a product (" cobalt–serum ") in which the cation was complexed with both proteins and small molecular components. In cultures of rat myoblasts, cobalt–serum was not only less toxic than the equivalent amount of ionic $Co^{2+}$, but also produced cytological changes similar to those observed in the vicinity of the implanted metal *in vivo*. It seemed therefore that, under the latter conditions, complexes of the dissolved metal with " biological ligands " might function in the transport of the cation into the free myoblasts, which are involved in the attempted repair of the damaged muscle and which, under the influence of the carcinogen, become malignant (Heath, 1960).

On this hypothesis, the initial solution of the metal becomes of particular importance. Further studies, the results of which are reported in this paper, have therefore been made on the dissolution of various carcinogenic and non-carcinogenic metals in horse serum, in other model systems and in muscle autolysates. The last of these seemed particularly relevant to the action of the carcinogenic metals, since these dissolve *in vivo* in a fluid environment, the main components of which are probably the breakdown products of the damaged muscle. Investigations have also been made on the effects of oxygen (previously noted by Heath *et al.*, 1969), on the solubility of cobalt in serum and other systems.

### MATERIALS AND METHODS

*Chemicals and biological materials.*—Sterile horse serum was obtained from the School of Veterinary Medicine, University of Cambridge. Penicillamine was a gift from Dista products, Speke, Liverpool; glutathione was purchased from British Drug Houses Ltd., Poole, Dorset; bovine albumin (Fraction V) from Armour Pharmaceutical Co. Ltd., Eastbourne; and triglycine from Sigma Chemical Co. Ltd., London, S.W.6.

Finely divided metallic cobalt was prepared by reduction of a solution of $CoCl_2 6H_2O$ (2 g) in 100 ml of 10 mmol/l acetic acid containing 1·59 g of $NaBH_4$. The black precipitate that separated was filtered with suction, washed with 10 mmol/l acetic acid and with water and dried *in vacuo*. The same procedure was used for the preparation of metallic nickel from $NiCl_2$ and $^{63}NiCl_2$. The latter was obtained from the Radiochemical Centre, Amersham, and was supplemented with carrier $NiCl_2$ to give a 0·01 mol/l solution that contained 1·45 $\mu$Ci $^{63}Ni^{2+}$/ml.

Commercial preparations of powdered metallic cadmium and zinc were obtained from Hopkins & Williams Ltd., Chadwell Heath, Essex, and British Drug Houses Ltd., London, respectively. All other metal powders were supplied by Johnson Matthey & Co. Ltd., London, E.C.1.

*Analytical procedures.*—Nitrogen was determined by the Kjeldahl method, diffusible peptides by the biuret reaction (Aldridge, 1957), lactic acid by the method of Barker and Summerson (1941) and amino acid with the ninhydrin reagent of Yemm and Cocking (1955). Metal analyses were made by atomic absorption with a Perkin Elmer Model 303 spectrophotometer. Protein solutions were digested before analysis with Aristar $HNO_3$, the blanks being treated similarly.

Oxygen consumption was measured in a Warburg apparatus, usually at 37° C, with air as the gas phase and a fluid volume of 2 ml. Aseptic conditions were obtained by the use of special flasks (Type AO5-65, Shandon Scientific Co. Ltd., London, N.W.10) which contained cotton filter pads below the ground glass joints. Metal powders were weighed into the flasks before sterilization, the serum or other solutions being added subsequently under aseptic conditions.

Paper chromatography was done on Whatman No. 1 paper and since metal complexes were to be separated, only neutral solvents were used. For one-dimension runs, the solvent was propan-1-ol-$H_2O$ (7 : 3). This solvent was also used as the first solvent, with water-saturated 2-methylbutan-2-ol as the second, in two-dimensional separations. Amino acids were located by spraying with 0·5% (w/v) ninhydrin in butan-1-ol, histidine being identified specifically on duplicate chromatograms with diazotized sulphanilic acid (Smith, 1958). Metals were detected by exposing the chromatogram to ammonia vapour and then spraying with a 1% (w/v) solution of rubeanic acid in ethanol. This reagent gave a blue colour with $Ni^{2+}$ and a yellow brown colour with $Co^{2+}$, the limit of detection being 0·03 $\mu$g of either cation.

*Preparation of metal serum complexes.*—The powdered metals (15 mg) were incubated aseptically with sterile horse serum (50 ml) at 37° C in 250 ml Erlenmeyer flasks, fitted with ground glass stoppers, or gas-washing-bottle heads. The latter had taps sealed on both arms. Aerobic and anaerobic incubations were performed with air and nitrogen, respectively, as the gas phase. The flasks were agitated gently at daily intervals. After termination of the reactions, the contents of the flasks were centrifuged and the supernatant fractions dialysed in Visking tubing against 2 or more changes of 10 volumes of 0·154 mol/l NaCl.

*Solution of cobalt metal in aqueous albumin.*—Cobalt powder (15 mg) was incubated aseptically with a 6·2% (w/v) solution of bovine serum albumin in 20 mmol/l phosphate buffer, pH 7·2 (15 ml) for 4 weeks at 37° C. The albumin solution alone as control was incubated for the same length of time under the same conditions. At the end of the incubation period a pink precipitate, identified by chemical analysis as $Co_3(PO_4)_2 8H_2O$, was present in the experimental flask. After centrifugation, both solutions were dialysed against 0·154 mol/l NaCl and analysed by the biuret method. After dialysis, the cobalt–albumin solution contained 0·76 μg $Co^{2+}$/mg protein.

*Reaction of cobalt metal with triglycine.*—Replicate samples (15 mg) of cobalt metal powder were incubated at 37° C in a series of sterile universal containers, each containing a solution (2 ml) of 0·1 mol/l triglycine, adjusted to pH 7·5 with NaOH, for 7 and 36 hours and for 4 days. At these times the solutions, which were deep pink in colour, contained 390, 675 and 1270 μg $Co^{2+}$/ml respectively. Samples (0·5 ml) of each were applied to a column (102 × 1·4 cm) of Sephadex G15 (Pharmacia (Great Britain) Ltd., London, W.5) and the components separated by gel filtration with water as eluant. Fractions (1 ml) were collected and analysed for $Co^{2+}$ and amino nitrogen.

*Reaction of ionic $Co^{2+}$ with triglycine.*—This was performed by the method of Gilbert, Otey and Price (1951). A sample of the product (1 ml; 240 μg $Co^{2+}$) at pH 7·4 was subjected to gel filtration on Sephadex G15, as described above.

*Solution of metals in tissue homogenates.*—The tissues were dissected under aseptic conditions and homogenized in Tyrode solution (10 ml/g wet weight tissue) with metallic cobalt, cadmium and nickel (15 mg) in a M.S.E. homogenizer fitted with a blending assembly (Cat. No. 77313) and sterile universal containers (Cat. No. 69344; Measuring & Scientific Equipment Ltd., London, S.W.1). The homogenates were incubated at 37° C, samples being removed at intervals and plated on nutrient agar as a check of sterility. Any preparations that showed bacterial contamination were discarded. After incubation (see " Results " section), the suspensions were centrifuged (20 minutes, 12,000*g*) and the supernatant fractions analysed for the appropriate cation. Part of each fraction was dialysed exhaustively against 0·154 mol/l NaCl for the determination of the distribution of the cation between the diffusible and non-diffusible molecules. This remainder was dialysed against water (3 × 200 ml) and the diffusates lyophilized. Approximately 50% of the soluble nitrogen of muscle homogenates was recovered in the diffusible components.

*Implantation of nickel-63 powder into rat muscle.*—Nickel-63 powder (5 μCi), suspended in horse serum (1·0 ml), was injected into each thigh muscle of six 2–3 month old female rats of the hooded strain, as described by Heath (1956). The animals were killed in pairs at daily intervals after injection, and muscle from the area of implantation dissected out. The tissue samples were homogenized in water, the homogenates centrifuged (10,000*g*, 20 minutes) and the supernatant fractions dialysed against water, the diffusates being lyophilized. For gel filtration, an aqueous solution (1 ml, 4·2 × $10^5$ counts/minute) of one of the products was run on a Sephadex G15 column with 25 mmol/l phosphate buffer, pH 7·2, as eluant. Portions (0·2 ml) of each fraction (1·0 ml) from the column were analysed for $^{63}Ni^{2+}$.

*Determination of $^{63}Ni^{2+}$.*—This was carried out as described by Webb and Weinzierl (1972).

*Biological effects of metal complexes.*—These were determined in cultures of embryonic rat myoblasts as described by Heath, Webb and Caffrey (1969).

### RESULTS

### *Solution of carcinogenic and non-carcinogenic metals in horse serum*

The carcinogenic metals nickel and cadmium dissolved slowly in horse serum at 37° but, in contrast to cobalt (Heath *et al.*, 1969), the solution of either was the same aerobically and anaerobically (Fig. 1). The $Ni^{2+}$ and $Cd^{2+}$ concentrations in nickel and cadmium sera were reduced by 50% and 80% respectively on dialysis against two changes of 10 volumes of 0·154 mol/l NaCl. Under the same conditions 70% of the $Co^{2+}$ in cobalt serum remained bound to the protein molecules.

Solubility in serum was not specific for the carcinogenic metals. Thus, although dissolution of iron in serum was insignificant (only 0·05 μg ions/ml after 28 days), zinc dissolved to give an approximately 0·5 mmol/l solution after 28 days under both aerobic and anaerobic conditions, about 30% of the dissolved $Zn^{2+}$ being removed by dialysis against 0·154 mol/l NaCl. The solubility of copper in horse serum, like that of cobalt, was

FIG. 1.—Solubility of cadmium (— — · —) and of nickel (- - - - -) metal powders in horse serum under aerobic (△, □) and anaerobic (▲, ■) conditions, in comparison with that of metallic cobalt (————) aerobically (○). Experimental details are given in " Materials and Methods ".

FIG. 2.—Effect of oxygen on the solution of metallic cobalt (—○—) and cadmium (- - ● - -) in triglycine. The powdered metals (50 mg) were incubated anerobically with a 20 mmol/l solution of triglycine (50 ml), adjusted to pH 7·4 with NaOH for 121 and 60 hours respectively, and then made aerobic.

FIG. 3.—Gel filtration on Sephadex G15 of the products formed (a) on solution of metallic cobalt in 0·1 mol/l triglycine at pH 7·5 and (b) by the interaction of ionic $Co^{2+}$ with triglycine at pH 10·15. The experimental details are given in the "Materials and Methods" section. Cobalt (—○—) was determined by atomic absorption and "peptides" (—●—) by the ninhydrin method, the results being expressed in arbitrary units (% absorption and $E_{626\ nm}$, respectively). Interference by the cation with the ninhydrin reaction was responsible for the apparent low concentrations of "peptide" in the $Co^{2+}$ peaks.

greatly increased in the presence of oxygen. Under aerobic conditions the metal dissolved with the formation of green solution, the $Cu^{2+}$ concentration of which (4·2 mmol/l after 28 days) was reduced by 25% on dialysis against 0·154 mol/l NaCl.

*The effect of oxygen on the solution of metallic cobalt in serum and other complexing agents*

As aids to the study of the complicated cobalt serum system, a number of simple models were used. In all of these, dissolution of cobalt aerobically was accompanied by the uptake of oxygen. Fig. 2 provides an illustration of the effect of oxygen on the solubility of metallic cobalt in a neutral 20 mmol/l solution of triglycine. In this experiment, the metal was incubated anaerobically with the peptide until equilibrium was reached (120 hours; 2·35 μg ions $Co^{2+}$/ml), and the system then made aerobic. Immediately solution of the metal recommenced, the content of the dissolved cation being increased by 224 % after a further 3 hours. Under the same conditions metallic cadmium at first dissolved rapidly and then, after 2–4 hours, more slowly in the triglycine solution, its solubility being unaffected by oxygen, whereas metallic nickel remained insoluble.

When cobalt metal was incubated aerobically with a neutral solution of triglycine under the conditions described in the " Materials and Methods " section, the cobalt dissolved rapidly to give a deep pink solution within a few hours. Gel filtration of the product on Sephadex G15 showed that cobalt was present in 3 fractions, and thus in at least 3 complexes of different molecular weights (Fig. 3a). One of these was identical with the cobalt triglycine complex (Fig. 3b) formed by the reaction of $CoCl_2$ with the tripeptide by the method of Gilbert, Otey and Price (1951). The formation of 2 additional

FIG. 4.—Oxygen uptake on solution of cobalt metal in solutions of glutathione. Finely divided metallic cobalt (1·5 mg) was incubated aseptically with (a) 2·27 mmol/l (—○—) and (b) 0·227 mmol/l glutathione (– –▲– –) in phosphate buffered saline (pH 7·7; 2·0 ml) in Warburg flasks at 37° C. Oxygen uptake was measured manometrically against blanks of the appropriate concentrations of glutathione alone. At the end of the incubation period (90 hours) the concentrations of dissolved $Co^{2+}$ in (a) and (b) were 40·0 and 2·4 μg/ml, respectively.

FIG. 5.—Uptake of oxygen by suspensions of powdered metallic cobalt, nickel and cadmium in phosphate buffered saline. The metals (1·5 or 15·0 mg) were incubated under aseptic conditions in phosphate buffered saline (pH 7·4; 2·0 ml). Oxygen uptake was measured manometrically. The metal powders that were used were cobalt, 1·5 mg (– –○– –) and 15·0 mg (—○—), and nickel, 15·0 mg (—□—) both prepared by chemical reduction of the corresponding chlorides, and commercial preparations of cobalt (1·5 mg, – –▲– –, and 15·0 mg —▲—), nickel (15·0 mg, —■—) and cadmium (15·0 mg – –▽– –). At the end of the incubation period (93 or 113 hours) the concentrations of dissolved cations in all systems were less than 1 μg/ml.

complexes on dissolution of metallic cobalt in triglycine was unlikely to be due to the difference in pH of the two systems, since the reaction of ionic $Co^{2+}$ with the peptide required the more alkaline conditions. Thus, some hydrolysis of the peptide bond appeared to accompany the solution of the metal. This was observed also when metallic cobalt dissolved in protein solutions. After incubation of cobalt powder (15 mg) with a phosphate (20 mmol/l, pH 7·2) buffered 6 % (w/w) solution of albumin for 28 days at 37°C, for example, the content of diffusible peptides was 7 % greater than in the control solution of albumin alone.

To determine whether sulphydryl compounds were particularly susceptible to catalytic oxidation by metallic cobalt, solubility of the latter in a solution of glutathione (2·27 mmol/l) was investigated. After 95 hours, however, when the concentration of dissolved $Co^{2+}$ was 0·67 mmol/l (Fig. 4), oxygen consumption (16 $\mu$g atoms/$\mu$mol glutathione) was in great excess of that required for oxidation to the disulphide. Furthermore, in a more dilute solution of glutathione (0·227 mmol/l), the solubility of the metal was decreased (to 0·04 mmol/l at 95 hours), although the rate of oxygen consumption was initially similar to that in the stronger solution (Fig. 4), whilst the total oxygen uptake (27·6 $\mu$g atoms oxygen), relative to the concentration of either glutathione or dissolved $Co^{2+}$, was increased. Since in these experiments the amount of metallic cobalt was constant, it seemed probable that the metal itself was continually absorbing oxygen.

Considerable oxygen absorption occurred when different amounts of cobalt powder were incubated in air in phosphate buffered saline. The extent of absorption was dependent on the quantity and particle size of the metal (Fig. 5), being greatest with the finely divided cobalt. After incubation, the residual cobalt powders appeared to have a slight green surface tinge. Powdered metallic nickel and cadmium showed negligible oxygen absorption, although some uptake occurred with finely divided nickel that was prepared by reduction of the chloride (Fig. 5). As observed previously (Heath *et al.*, 1969) metallic cobalt, cadmium and nickel are essentially insoluble in phosphate buffered saline and, in the present experiments, the concentrations of free cations in solution were less than 1 $\mu$g/ml.

Finely divided metallic cobalt, obtained by chemical reduction of $CoCl_2$ (see " Materials and Methods " section) dissolved more rapidly in serum than did the commercial preparations that were used previously (Heath *et al.*, 1969), solution being accompanied by a high rate of oxygen consumption (*e.g.* 38·2 $\mu$g atoms oxygen/ml serum after 95 hours). Although this appeared to be independent of the concentration of $Co^{2+}$ in solution (*i.e.* 2·85 and 4·25 $\mu$g ions/ml) Fig. 6), the slow, essentially insignificant oxygen consumption by serum alone was increased to 6·4 $\mu$g atom/ml by the addition of either ionic $Co^{2+}$ or the " cobalt–serum " complex (Fig. 7). Thus, some catalytic oxida-

FIG. 6.—Oxygen uptake on solution of cobalt metal in horse serum. Finely divided metallic cobalt (1·5 mg) prepared by reduction of $CoCl_2$, was incubated at 37° C in each of 2 Warburg flasks with sterile horse serum (2·0 ml). Oxygen consumption was measured manometrically over a period of 95 hours. After this time the concentration of $Co^{2+}$ in solution in flask 1 (—○—) was 171 $\mu$g/ml and in flask 2 (- -▲- -) 255 $\mu$g/ml.

FIG. 7.—Oxidation of serum produced by either $Co^{2+}$ ions, or the cobalt–serum complex. (a) Ionic $Co^{2+}$. Sterile solutions (0·25 ml) of $CoCl_2$ in 0·9% (w/v) NaCl were added aseptically to horse serum (2·0 ml) in Warburg flasks to give final $Co^{2+}$ concentrations of 45·5 μg/ml (--▲--) and 89·0 μg/ml (—△—). The blank flask contained 0·25 ml of saline and 2·0 ml of serum. (b) Cobalt–serum. Volumes of 0·5 ml (—●—) and 0.2 ml (—○—) of a preparation of cobalt–serum (171 μg $Co^{2+}$/ml) were added aseptically to Warburg flasks that contained 1·5 ml and 1·8 ml of horse serum, the final concentrations of bound $Co^{2+}$ being 42·8 and 17.1 μg/ml respectively. The blank flask contained 2.0 ml serum. Oxygen uptake was followed manometrically for 106 hours at 37°C.

tion of the organic components of the serum by the dissolved cation was probable although, as in the more simple systems, the main uptake of oxygen occurred through adsorption of oxygen by the metal powder, or by oxide on its surface.

*Solubility of cobalt, nickel and cadmium in tissue homogenates*

Implants of radioactive nickel powder dissolved rapidly in rat skeletal muscle *in vivo*. Twenty-four hours after implantation, the muscle appeared macroscopically normal and nickel powder was still present at the injection site. After 3 days, however, the metal had disappeared and the muscle was haemorrhagic. In homogenates of muscle that were prepared at 1, 2 and 3 days after implantation of the radioactive nickel, about 80% of the dissolved $^{63}Ni^{2+}$ was found to be distributed among the small diffusible tissue components. When nickel metal was incubated with a sterile homogenate of rat muscle in Tyrode solution (1 g tissue/10 ml Tyrode), a concentration of about 1·4 mmol/l $Ni^{2+}$ was obtained in the soluble fraction after 3 weeks. On dialysis of this fraction against 15 volumes of water, over 90% of the nickel was recovered in the diffuse. Gatel filtration of a sample of these small diffusible molecules from both the *in vivo* and *in vitro* experiments gave a similar pattern of nickel distribution (Fig. 8). Cobalt dissolved readily in a rat muscle homogenate (over 5 μg ions/ml in 2 weeks), its solubility in this system being greater than in horse serum. Other metals (copper, cadmium and zinc) also dissolved under these conditions, the solubility of cadmium being about 0·6 mmol/l in 2 weeks. As with cobalt, over 90% of the dissolved cations were bound to the small diffusible molecules.

The 3 carcinogenic metals also dissolved in homogenates of other tissues (Table I). Cobalt and nickel both dissolved to the same extent in homogenates of rat liver, heart and kidney. Cadmium, however, dissolved only slowly in suspensions of rat heart and kidney, but very rapidly in homogenates of the liver. In all preparations, about 95% of the dissolved metal was bound by the small diffusible molecules.

*Exchange reactions*

When dialysed cobalt serum (12 ml; 2 μg ions $Co^{2+}$/ml) was added to a solution of penicillamine (2 mg/ml) in 50 mmol/l tris buffer, pH 7·2, there was an immediate exchange of $Co^{2+}$, as shown by an instantaneous colour change to yellow. On

Fig. 8.—Gel filtration on Sephadex G15 of the diffusible products from the dissolution of metallic nickel-63 in rat muscle *in vivo* (– –●– –) and of metallic nickel in a muscle homogenate *in vitro* (—○—). For experimental details see " Materials and Methods ". The samples applied to the column contained 4·2 × $10^5$ counts/minute $^{63}Ni^{2+}$ and 175·5 $\mu g$ $Ni^{2+}$ iespectively.

subsequent dialysis, about 60 % of the cobalt was recovered bound to the diffusible penicillamine molecules. Addition of dialysed cobalt serum (6 ml, 2 $\mu g$ ions $Co^{2+}$/ml) to a solution of the diffusible components of a rat muscle autolysate (8 ml, 1 mg N/ml) also resulted in the redistribution of the cation, 60 % of which became bound to the small molecules. These values were not altered significantly after incubation of the mixtures, sterilized by filtration through a Millipore (0·45 $\mu m$ pore size) membrane, for up to 18 days. In the reverse experiment, 85–90 % of the cobalt remained bound to the small molecules of rat muscle diffusate (6 ml, 4·8 $\mu g$ ions $Co^{2+}$/ml) when the latter was mixed with dialysed serum (6 ml, 10 mg N/ml).

*Distribution of cobalt and nickel among the diffusible components of rat muscle homogenate*

At least 8 ninhydrin-positive spots, of

TABLE I.—*Solution of Metallic Cobalt, Cadmium and Nickel in Homogenates of Rat Tissue*

Homogenates of the different tissues were prepared in Tyrode solution (1 g wet weight tissue/10 ml) and incubated with each of the 3 metals as outlined in " Materials and Methods ". After incubation at 37° C for 18 days*, the suspensions were centrifuged (20 minutes, 12 000*g*) and the supernatant fractions analysed for the appropriate cation. Portions of each were dialysed exhaustively against 0·154 mol/l NaCl and the non-diffusible fractions also analysed. The percent diffusible cation was calculated from these results, after correction for any volume change during dialysis.

| | Soluble fraction from homogenate of | | | | | | | |
|---|---|---|---|---|---|---|---|---|
| | Muscle | | Liver | | Heart | | Kidney | |
| | Cation concentration (mmol/l) | Diffusible cation (%) | Cation concentration (mmol/l) | Diffusible cation (%) | Cation concentration (mmol/l) | Diffusible cation (%) | Cation concentration (mmol/l) | Diffusible cation (%) |
| Cobalt . . | 5·06 | 90 | 2·26 | 98 | 2·24 | 96 | 2·17 | 98 |
| Cadmium . | 0·53 | 90 | 4·39 | 98 | 0·25 | 91 | 0·33 | 97 |
| Nickel . . | 1·45 | 98 | 2·52 | 98 | 2·18 | 97 | 1·93 | 99 |

* Homogenates of muscle were incubated with metallic cobalt for 14 days, and with the metals, nickel and cadmium, for 21 days.

$R_F$ values from 0·075 to 0·6, were detected in normal muscle diffusate, cobalt–muscle diffusate and nickel–muscle diffusate, by paper chromatography for 16 hours with propan-1-ol-$H_2O$ as solvent. Certain of these were resolved into 2 or 3 components by two-dimensional chromatography with 2 methyl-butan-2-ol as the second solvent. As, in both the nickel– and cobalt–muscle diffusates, the cations were associated with all ninhydrin-positive components, identification of the individual amino acids (and peptides) was not attempted. In each chromatogram, however, a particularly high concentration of the cation was bound by a single component, characterized as histidine. Significant amounts of both metals also remained at the origin and occurred at $R_F$ 0·4 (in propan-1-ol-$H_2O$). Neither of these areas contained amino acids, but both had absorbance in the u.v. region ($\lambda_{max}$ 244 and 265 nm, and 268 nm, respectively) and probably contained mixtures of nucleotides, and nucleosides or free bases.

Although the muscle diffusate contained large amounts of lactate (4–5 mg/g wet weight original tissue), this seemed of minor importance in relation to the dissolution of the carcinogens. Metallic cobalt, for example, dissolved when incubated with sterile 0·33 mol/l sodium lactate in Tyrode solution (5·2 $\mu$g ions $Co^{2+}$/ml after 14 days), but metallic nickel remained insoluble.

### *Effects of metal complexes in cultures of rat myoblasts*

In actively growing cultures of rat myoblasts " nickel serum " and " nickel–muscle-diffusate ", at concentrations to give 10 $\mu$g $Ni^{2+}$/ml, and " cobalt–muscle-diffusate " ($\equiv$ 3·3–5·0 $\mu$g $Co^{2+}$/ml medium) produced cytological changes similar to those produced by cobalt–serum (Heath *et al.*, 1969), the alterations in nuclear morphology being particularly conspicuous after 12–14 days. The biological effects of the corresponding complexes of cadmium have not been determined.

### DISCUSSION

In previous work (Heath *et al.*, 1969) it was found that the carcinogenic metal, cobalt, dissolves when incubated aseptically with horse serum to yield a solution in which the $Co^{2+}$ cation is complexed with both proteins and small molecular weight components. In contrast to the free $Co^{2+}$ cation, this cobalt–serum is not only less toxic for myoblasts *in vitro*, but also produces in these cells cytological changes similar to those seen in the neighbourhood of metal implants *in vivo*.

The present results show that in addition to cobalt, various metals, including the carcinogens, nickel and cadmium, also dissolve in horse serum. With the exception of copper, dissolution of these metals, unlike that of cobalt, is unaffected by the absence of oxygen.

In aqueous media it is probable that particles of finely divided metals are covered with surface films of oxides, hydroxides and/or basic carbonates and sulphates. Initially, therefore, the solubility of a metal in serum or other complexing agent could depend amongst other factors on the solubility products of its surface components. The rate of solution of cadmium in triglycine (Fig. 2) shows an abrupt discontinuity after about 4 hours, which may indicate the removal of a more soluble surface film. If solution of metals in biological fluids occurs *via* the intermediary formation of hydrated oxides for example, the concentrations of $Co^{2+}$, $Ni^{2+}$ and $Fe^{3+}$ in equilibrium with the corresponding metals at pH 7·0 would be $1{\cdot}6 \times 10^{-4}$ mol/l, $8{\cdot}9 \times 10^{-5}$ mol/l and $3{\cdot}8 \times 10^{-17}$ mol/l, since the solubility products of $Co(OH)_2$, $Ni(OH)_2$ and $Fe(OH)_3$ are $1{\cdot}6 \times 10^{-18}$, $8{\cdot}9 \times 10^{-19}$ mol/l and $3{\cdot}8 \times 10^{-38}$ mol/l, respectively. Addition of an agent (*e.g.* protein) which chelates the free cation, disturbs the equilibria and the surface hydroxides dissolve. On the assumption that the velocity constants ($K_F$) for the forward reaction

$$M^{n+} + \text{protein} \xrightarrow{K_F} M^{n+}\,\text{protein} + nOH^-$$

(or $nH_2O$, if chelation involves the elimination of $H^+$)

do not vary by more than a few orders of magnitude, then the rate of solution (*i.e.* $K_F\,[M^{n+}]\,[\text{protein}]$) would be much greater for either $Co^{2+}$ or $Ni^{2+}$ than for $Fe^{3+}$ and thus, as observed experimentally, metallic cobalt and nickel would dissolve in serum at an appreciable rate, whereas iron would not.

Solution of soluble metals would be increased under conditions that, once the surface film had been removed, the under-lying metal was re-oxidized. The increased solubility of both cobalt and copper in serum under aerobic conditions could be due to re-oxidation of the metal surface, which would depend upon its physical and chemical properties. In this connection, it may be significant that the solubility in serum of finely divided metallic nickel, produced by the chemical reduction of $NiCl_2$, is increased in the presence of oxygen, whereas that of the coarser, commercial preparation is not. Such reasoning, however, is inadequate to explain all the experimental facts and in particular fails to account for the insolubility of nickel in a neutral solution of triglycine, in which cobalt and cadmium dissolve readily. At present, therefore, there is no satisfactory explanation for the mechanisms of solution of metals in biological fluids such as serum. Undoubtedly, the process is complex and may involve a number of contributory reactions. Perlstein, Alassi and Cheng (1971), for example, have observed that preparations of Raney nickel (including those made by reduction of $(CH_3COO)_2Ni$ with $NaBH_4$), under defined conditions, cause desulphurization of both free and protein-bound cysteine to alanine. A similar reaction may occur at least with cobalt powder, since Heath *et al.* (1969) observed that granules of the metal, which remained undissolved after incubation with horse serum, evolved $H_2S$ on treatment with dilute HCl.

Although metallic cobalt powder does not dissolve when incubated in phosphate buffered saline in the absence of a chelating agent, it takes up a large amount of oxygen and, after completion of the reaction, often appears to have a brown or green tinge on its surface. Cobalt oxides are known to adsorb oxygen superficially to a composition of $Co_3O_4$ and beyond, whilst $Co(OH)_2$ can form CoO.OH. Solution of either of these in serum would give rise to complexes of trivalent cobalt. In addition, it is also possible that $Co^{2+}$ complexes in solution may be oxidized to form peroxo-bridged compounds or, more probably the stable $Co^{3+}$ complexes. Such reactions would contribute both to the oxygen uptake that is observed when the metal dissolves aerobically in serum and also to the increased solubility of cobalt in the presence of air. One of the features of the chemistry of cobalt is the very ready formation of $Co^{3+}$ complexes in the presence of chelating agents and molecular oxygen. These complexes, which are considerably more stable than are those of $Co^{2+}$, can be formed from the latter in 2 ways:

$$(1)\ CoL_6^{2+} + \tfrac{1}{4}O_2 + \tfrac{1}{2}H_2O \rightarrow CoL_6^{3+} + OH^-$$

$$(2)\ CoL_6^{2+} + O_2 + H^+ \rightarrow CoL_6^{3+} + .O_2H$$

Several examples of the second reaction which results in the production of free radicals, are known, and these always involve the oxidation with molecular oxygen of a metal variable valency (Harris, Herp and Pigman, 1971). Formation of free radicals in this way is known to cause cleavage of polysaccharides (Harris *et al.*, 1971) and it is possible that in the cobalt–serum system a similar process is responsible for the hydrolysis of peptide bonds, as has been observed in solutions of proteins (*e.g.* albumin) and a simple peptide (*e.g.* triglycine, Fig. 3).

Although the mechanisms of solution of the metals in tissue homogenates may be basically similar to those involved in the serum systems, the processes may be modified by the fact that, at least in freshly prepared homogenates, biological oxidations and reductions occur and there is a continual turnover of electron acceptors. Usually, cobalt is more soluble than nickel or cadmium, but there is some tissue specificity since, in homogenates of liver, cadmium is the most soluble of the 3 metals.

In homogenates of muscle, as in those of other tissues, dissolved cobalt, cadmium and nickel ions are bound by the small diffusible molecules, rather than by the protein components. The same seems to be true when the metals dissolve *in vivo*, since the products of the solution of nickel-63 in the thigh muscle of the living animal are similar to those obtained when the metal is incubated with an homogenate of the tissue *in vitro*. The metal ions are not bound by a single specific component, but are distributed among a number of compounds. This distribution appears to be comparable at least for nickel and cobalt. These observations, coupled with the results of the exchange reactions, which show that the small molecular components of a sterile autolysate have a greater affinity for dissolved metals than serum proteins, suggest that when the metal ions are incorporated into cells *in vivo*, these diffusible complexes are the normal carriers of the cations. In both serum and muscle homogenates, solubility is not confined specifically to the carcinogenic metals, as other, non-carcinogenic metals also dissolve. The specificity of the former metals, therefore, would appear to reside in the subsequent effects of the dissolved cations, once these are incorporated intracellularly.

This work was supported by the Medical Research Council. The authors thank Mr G. Payton for his assistance and cooperation in the experiments that involved the implantation of metallic nickel into live rats.

### REFERENCES

Aldridge, W. N. (1957) Liver and Brain Mitochondria. *Biochem. J.*, **67**, 423.

Barker, S. B. & Summerson, W. H. (1941) The Colorimetric Determination of Lactic Acid in Biological Materials. *J. biol. Chem.*, **138**, 535.

Gilbert, J. B., Otey, M. C. & Price, V. E. (1951) The Enzymatic Susceptibility of the Red Cobalt Complexes of Several Dipeptides. *J. biol. Chem.*, **190**, 377.

Harris, M. J., Herp, A. & Pigman, W. (1971) Depolymerization of Polysaccharides Through the Generation of Free Radicals at a Platinum Surface. A Novel Procedure for the Controlled Production of Free Radical Oxidations. *Archs Biochem. Biophys.*, **142**, 615.

Heath, J. C. (1956) The Production of Malignant Tumours in the Rat by Cobalt. *Br. J. Cancer*, **10**, 668.

Heath, J. C. (1960) The Histogenesis of Malignant Tumours Induced by Cobalt in the Rat. *Br. J. Cancer*, **10**, 478.

HEATH, J. C. & DANIEL, M. R. (1964*a*) The Production of Malignant Tumours in the Rat by Cadmium. *Br. J. Cancer*, **18**, 124.

HEATH, J. C. & DANIEL, M.A. (1964*b*) The Production of Malignant Tumours by Nickel in the Rat. *Br. J. Cancer*, **18**, 261.

HEATH, J. C., DANIEL, M. R., DINGLE, J. T. & WEBB, M. (1962) Cadmium as a Carcinogen. *Nature, Lond.*, **193**, 592.

HEATH, J. C., WEBB, M. & CAFFREY, M. (1969) Interaction of Carcinogenic Metals with Tissue and Body Fluids. Cobalt and Horse Serum. *Br. J. Cancer*, **23**, 153.

PERLSTEIN, M. A., ALASSI, M. Z. & CHENG, S. M. (1971) Desulfurization of Sulfur Amino Acids and Proteins with Raney Nickel. *Biochim. biophys. Acta*, **236**, 174.

SMITH, I. (1958) *Chromatographic Techniques: Clinical and Biochemical Applications*. London: Heinemann. p. 145.

WEBB, M. & WEINZIERL, S. M. (1972) Uptake of $^{63}Ni^{2+}$ from Its Complexes with Proteins and Other Ligands by Mouse Dermal Fibroblasts *in vitro*. *Br. J. Cancer*, **26**, 292.

YEMM, E. W. & COCKING, E. C. (1955) Determination of Amino Acids with Ninhydrin. *Analyst*, **80**, 209.